# Tear biomarkers for Alzheimer’s disease screening and diagnosis (the TearAD study): design and rationale of an observational longitudinal multicenter study

**DOI:** 10.1186/s12883-023-03335-y

**Published:** 2023-08-05

**Authors:** Nienke van de Sande, Inez H. G. B. Ramakers, Pieter Jelle Visser, Frans R. J. Verhey, Frank D. Verbraak, Femke H. Bouwman, Tos T. J. M. Berendschot, Rudy M. M. A. Nuijts, Carroll A. B. Webers, Marlies Gijs

**Affiliations:** 1 School of Mental Health and Neuroscience (MHeNs), University Eye Clinic Maastricht, Maastricht University, Maastricht, The Netherlands; 2 Department of Psychiatry and Neuropsychology, Alzheimer Center Limburg, School of Mental Health and Neuroscience (MHeNs), Maastricht University, Maastricht, The Netherlands; 3 Alzheimer Center Amsterdam, Department of Neurology, Amsterdam University Medical Center, Amsterdam, Netherlands; 4 Ophthalmology Department, Amsterdam UMC, location VUmc, Amsterdam, The Netherlands

**Keywords:** Alzheimer’s disease, Tear fluid, Retinal nerve fiber layer, Amyloid beta, Neurofibrillary tangles, Cognition, Dementia, Biomarkers

## Abstract

**Background:**

Alzheimer’s disease (AD) is the most common cause of dementia, and due to increasing life expectancy the number of patients is expected to grow. The diagnosis of AD involves the use of biomarkers determined by an amyloid PET scan or cerebrospinal fluid analyses that are either invasive or expensive, and not available in each hospital, thus limiting their usage as a front-line screener. The TearAD study aims to use tear fluid as a potential source for AD biomarkers. In previous reports, we demonstrated that AD biomarkers amyloid-beta and tau, are measurable in tear fluid and are associated with disease severity and neurodegeration. This study aims to validate previous results in a larger cohort and evaluate the diagnostic accuracy of tear biomarkers to discriminate between individuals with and without neurodegeneration as determined by hippocampal atrophy.

**Methods:**

The TearAD study is an observational longitudinal multi-center study that will enroll 50 cognitively healthy controls, 50 patients with subjective cognitive decline, 50 patients with mild cognitive impairment and 50 patients with AD dementia from the memory clinic. Participants will be examined at baseline, after one year, and after two years follow-up. Study assessments include neuropsychological tests and ophthalmic examination. All participants will receive a MRI scan, and a subset of the study population will undergo cerebral spinal fluid collection and an amyloid PET scan. Tear fluid will be collected with Schirmer strips and levels of Aβ38, Aβ40, Aβ42, t-tau and p-tau in tear fluid will be determined using multiplex immunoassays. Blood samples will be collected from all participants. Images of the retina will be obtained with a standard, hyperspectral and ultra-wide field fundus camera. Additionally, macular pigment optical density will be measured with the macular pigment reflectometer, and cross-sectional images of the retina will be obtained through optical coherence tomography imaging.

**Discussion:**

The TearAD study will provide insight into the potential diagnostic use of tear biomarkers as a minimally invasive and low cost tool for the screening and diagnosis of AD.

**Trial registration:**

Retrospectively registered at clinicaltrials.gov (NCT05655793).

## Background

Approximately 57 million people are affected by dementia globally, and due to the ageing of the global population this number is expected to increase to 152 million cases by the year 2050 [1]. Patients with dementia experience memory problems, disorientation and difficulties with performing routine tasks. Alzheimer’s disease (AD) is the most common cause of dementia. AD is associated with the accumulation of senile plaques (deposits of amyloid-beta (Aβ)) and neurofibrillary tangles (deposits of tau protein (NFT)) in the brain [2] as well as brain atrophy in the cerebral cortex and hippocampus. Other pathological processes can be observed in AD patients alongside or caused by the accumulation of Aβ-plaques and NFT, such as inflammation, oxidative damage, and blood-brain barrier dysfunction [3].

Timely diagnosis is important to promote early and optimal management of the disease. Furthermore, this will be essential for future therapies as they may be more effective when started earlier in the disease process. Patients are diagnosed with subjective cognitive decline (SCD), mild cognitive impairment (MCI) or AD dementia based on clinical symptoms, cognition and daily functioning [4–7]. Additionally, the etiology of the disease can be determined with the use of biomarkers. These biomarkers are classified into three categories: Aβ deposition, neurofibrillary tangles and neurodegeneration (ATN) [8]. Cerebral spinal fluid (CSF) analysis or amyloid positron emission tomography (PET) imaging are currently used for biomarker determination [5, 6]. However, these diagnostic tests are invasive, expensive and not widely available, thereby limiting their potential as a front-line screener. Considering the expected increase of people affected by AD, there will be a growing need for non-invasive and cost-effective tools allowing identification of patients in the earlier phases of the disease [1].

In this study, we propose using tear and retinal biomarkers to mirror changes in the brain. The eyes and the brain are closely connected through the optic nerve and both the retina and the central nervous system share a common origin from the developing neural tube. Given this relation, many brain diseases have ocular manifestations [9].

Retinal abnormalities are observed in AD patients and with the advent of high resolution imaging modalities these retinal measures can be quantified in vivo. Decreased peripapillary retinal nerve fiber layer (RNFL) and macular thickness is observed in patients with AD dementia and MCI [10]. Specifically, in patients with AD type dementia a decreased choroidal thickness is observed [11]. Another interesting parameter is the macular pigment optical density (MPOD). MPOD is a biomarker for the amount of pigment in the brain, mainly lutein, which has shown a strong correlation with cognition [12]. Fundus photography can provide information about the retinal vasculature. MCI and AD dementia patients display significant differences in the retinal vasculature of their peripheral retina regions when examined through ultra-wide field imaging [13]. Additionally, the foveal avascular zone in AD dementia patients was significantly larger [11]. All of the retinal imaging parameters combined can provide a total retinal fingerprint with an array of variables that uncover the functionality of using retinal changes as a biomarker for AD.

The main interest of this study is the biomarkers extracted from tear fluid. Tears covering the ocular surface are an important bio-fluid containing thousands of molecules, including proteins, lipids, metabolites, nucleic acids, vitamins and electrolytes [14]. Tear fluid is produced by the lacrimal gland, Meibomian glands and Goblet conjunctival cells and has an important role in preserving the healthy functioning of the eyes (e.g. washing away debris and irritants but also providing white blood cells in case of injury) [14]. Protein secretion in the lacrimal gland is highly regulated by both parasympathetic and sympathetic innervation [15]. We recently demonstrated that Aβ38, Aβ40 and Aβ42, total-tau (t-tau) and phosphorylated-tau (p-tau) are detectable in tear fluid, whereas tear p-tau levels were only detectable in SCD, MCI and AD dementia patients, but not in cognitively healthy controls [16]. Additionally, tear t-tau levels were significantly elevated in AD dementia patients compared to SCD patients [17]. More recently, a paper from Gharbiya and colleagues was able to reproduce the detection of p-tau and Aβ42 in the tear fluid of MCI and AD dementia patients [18]. In their study, levels of Aβ42 in tear fluid were significantly lower in MCI and AD dementia patients compared to cognitively healthy controls.

The TearAD study continues to investigate the potential of tear and retinal biomarkers in the screening and diagnosis of AD. To address this, we established a large observational multicenter study including 200 participants who will be followed for 2 years. The study design is described in detail in anticipation of upcoming study results.

## Methods

### Objectives

The primary objective of this study is to evaluate diagnostic accuracy measures of tear biomarkers to discriminate participants with and without neurodegeneration as determined by hippocampal atrophy. The secondary objective is to determine the level of agreement between Aβ and tau protein levels in tear fluid, blood and CSF or PET. Thirdly, we aim to investigate whether the combination of retinal biomarker with tear biomarkers can aid in determining the presence of neurodegeneration as determined by hippocampal atrophy. The final objective is to analyze how biomarkers in tear fluid alter over the course of the follow-up period.

### Study design and setting

This study is an observational longitudinal multicenter study that will be performed at the Maastricht University Medical Center (Maastricht UMC+) and the Amsterdam University Medical Center (Amsterdam UMC) in the Netherlands.

At Maastricht UMC+, people with cognitive complaints who are referred to the memory clinic are asked to participate in ongoing research. If they agreed on being contacted for clinical studies and have the diagnosis SCD, MCI or dementia due to AD they are asked to participate in the present TearAD study. In addition, community-based cognitively healthy controls were recruited through advertisement as part of the GlyM study and later asked to participate in the TearAD study (NL72269.068.19, Glymphatic dysfunction in cognitive impairment: a memory clinic study). Overlapping study procedures will not be repeated, but data will be shared between both studies for which permission granted through the study protocol and informed consent.

At Amsterdam UMC, people with cognitive complaints and a diagnosis of SCD, MCI or AD dementia will be included in the MHIRA study (NL70896.029.19, The diagnostic value of metabolic hyperspectral imaging of the retina in Alzheimer’s disease). These participants originate from the memory clinic based Amsterdam Dementia Cohort [19, 20]. Cognitively healthy controls will be recruited from the Innovative Medicine Initiative European Medical Information Framework for AD (EMIF-AD) PreclinAD study [21]. Agreements on sharing material and data are covered by a material and data transfer agreement between both parties.

Participants for the TearAD study are included from different cohorts with similar study procedures, but different primary endpoints and available infrastructure. For instance, amyloid PET imaging is available in Amsterdam UMC but not in Maastricht UMC+. For this reason, some of our secondary outcome measures will be analyzed in a sub-cohort of the total study population.

### Study population

Based on their clinical diagnosis we aim to include 50 patients with SCD, 50 patients with MCI and 50 patients with AD dementia. Additionally, 50 cognitively healthy controls will be included. Eventually, the total study population (*n* = 200) will consist of two groups of 100 participants with signs of neurodegeneration and 100 participants without signs of neurodegeneration. The presence of neurodegeneration will be defined as presence of medial temporal lobe atrophy (MTA) (measured by MRI using visual rating scores of the medial temporal lobes using the Scheltens Visual Rating Method abnormal cutoff of ≥ 2 [22]). The inclusion criteria are described in Tables 1 and 2.

**Table 1:** Inclusion criteria for all participants

**Inclusion criteria for all participants**
Available CSF, PET, CT or MRI data to evaluate the presence/absence of neurodegeneration (preferably within 1 year of inclusion in this study)
Absence of ocular conditions that could influence tear biochemical parameters (including eye infection, eye inflammation, eye surgery within the last 28 days or other acute eye conditions)
Absence of neurological or systemic chronic conditions known to interfere with retinal thickness (e.g. glaucoma, diabetes mellitus)
Absence of ocular conditions interfering with OCT quality/retinal thickness: e.g. severe cataract, age-related macular degeneration, and glaucoma
Written informed consent obtained and documented
Age > 50 years
No indications that follow-up (up to 24 months) is not possible

**Table 2:** Additional inclusion criteria

**Additional inclusion criteria for cognitively healthy controls only**
MMSE score 26–30 at baseline
Absence of cognitive complaints or treatment and did not seek help for cognitive complaints in the present moment or the past
**Additional inclusion criteria for patients only (SCD/MCI/AD Dementia)**
Capable of giving informed consent (MMSE score > 17/30)

The clinical diagnosis of dementia will be made based on the Diagnostic and Statistical Manual of Mental Disorders-5 (DSM-5) criteria for major neurocognitive disorders in Maastricht UMC + and the Amsterdam UMC the clinical National Institute on Ageing-Alzheimer’s Association (NIA-AA) criteria [4, 6, 23]. Patients with dementia have significant cognitive decline and this results in interference with independence in daily functioning. The clinical diagnosis of MCI will be made according to the clinical NIA-AA criteria; MCI patients have impaired cognitive functioning but preserved functioning in daily living [5]. Patients will be classified as SCD when they present to the memory clinic with cognitive complaint initiation, but no impairment in cognitive test performances and activities of daily living can be objectified [7]. Participants with absence of cognitive complaints or treatment and who did not seek help for cognitive complaints in the past will be grouped as cognitively healthy controls.

### Outcome measures

1. Relation between tau protein levels in tear fluid and neurodegeneration determined by hippocampal atrophy.
2. The level of agreement between Aβ biomarkers in tear fluid and Aβ positivity as determined by PET or CSF.
3. The level of agreement between Aβ and tau protein levels in tear fluid, blood and CSF.
4. The combination of retinal biomarkers and tear biomarkers as a predictor for neurodegeneration determined by hippocampal atrophy.
5. Mean change in tear biomarkers between baseline, 12 months and 24 months.

### Study assessments

Table 3 presents an overview and timing of the assessments. Follow-up visits are scheduled after 12 and 24 months.

**Table 3:** Overview and timing of study assessments

Assessment	Baseline	12 months	24 months
In-/exclusion criteria	X		
Informed consent	X		
Medical history and medication	X	X	X
Routine neuropsychological tests^a^	X	X	X
CT or MRI scan^b^	X		
PET scan or CSF sampling^c^	X		
Blood sampling^d^	X		X
Routine ophthalmic examination^e^	X	X	X
Tear sampling^f^	X	X	X
Fundus imaging^g^	X	X	X
OCT imaging^h^	X	X	X

^a^ Neuropsychological assessment will be performed according to local routine protocol of memory clinic, as described elsewhere [19]. Covering the cognitive domains of global cognition, memory, attention, executive functioning and language

^b^ Images will be acquired with a 3 Tesla MRI-scan and 7 Tesla MRI for a small sub-cohort. The Scheltens MTA visual rating scale will be used to score each hemisphere (range 0–4) [22]. A summed score of both hemispheres will be defined abnormal with a cutoff of ≥ 2 [24]

^c^ CSF fluid will be available from a subset of participants and will be analyzed for Aβ 42, t-tau and p-tau-181 using an immunoassay, local cut-off values for each clinic will be used [20, 25]. A PET scan will also be available from a subset of participants, from which standard uptake value ratio (SUVr) images will be generated and visually read by an experienced nuclear physician. Images will be classified as positive or negative rating for Aβ according to criteria defined by the manufacturer (GE Healthcare) [26]

^d^ Participants will undergo venipuncture for blood sampling and subsequent analysis of Aβ42/40, t-tau and p-tau AD biomarker levels using immunoassays

^e^ The presence of potential ocular pathologies or unexpected findings will be assessed during the standard ophthalmic examination during which we will analyze visual acuity (using the Early Treatment Diabetic Retinopathy Study (ETDRS) chart (27)), (auto-) refraction, intraocular pressure (using non-contact tonometry), axial length (IOLMaster™, Carl Zeiss Meditec AG, Jena, Germany) and general eye health (using slit lamp biomicroscopy and funduscopy)

^f^ Tear sampling will be performed by absorption of tear fluid in a paper Schirmer’s strip (TEAR strips, Contacare Ophthalmics and diagnostics, Gujarat, India). The Schirmer’s strip will be placed in de lower eyelid of both eyes. After 5 min, the Schirmer’s strip will be gently removed using tweezers and stored at -80 °C. Tear fluid will be extracted from Schirmer’s strips [27], and analyzed with targeted immuno-assays for Aβ 42/40, t-tau and p-tau

^g^ Fundus imaging captures images of the retina, optic nerve head, macula, retinal blood vessels, choroid, and the vitreous. We will use a the Clarus 700 fundus camera (Carl Zeiss Meditec AG, Jena, Germany) to capture images of the central retina. Color and autofluorescence ultra-wide field images of 200 degrees of the peripheral retina will be acquired using the California_rg_ fundus camera (Optos, Dunfermline, United Kingdom). Additionally, hyperspectral retinal imaging will be performed using the hyperspectral camera Optina-4 C (Optina Diagnostics, Quebec, Canada). Optina-4 C is a non-CE marked mydriatic camera combined with a tunable filter, thereby only permitting selected wavelengths (energy spectrum between 450 and 900 nm) [28]. In a subset of participants the macular pigment optical density will be determined in the right eye with the macular pigment reflectometer [29]

^h^ Optical coherence tomography (OCT) imaging will generate cross sectional retinal images from the (papillary) retinal nerve fiber layer thickness (RNFL) along with other parameters will be extracted. For each eye the following scans will be made, (i) fast enhanced depth imaging retina macula scan, (ii) dense macular scans, (iii) axonal ring scan around the optic nerve head (ONH), and (iv) an ONH scan using spectral domain OCT. Additionally, an OCT angiography (OCTA) will be done in a subset of the study population (Heidelberg Engineering, Heidelberg, Germany)

### Sample size

Sample size is based on our main outcome measure, which is estimating the diagnostic accuracy of biomarkers in tear fluid to discriminate between participants with and without neurodegeneration as determined by hippocampal atrophy. The calculation of sample size is based on the width of the 95% confidence intervals of sensitivity and specificity, as well as recent pilot data from our group [16]. To obtain a margin of error of maximally 10% for sensitivity as well as specificity, 97 individuals with neurodegeneration and 97 without neurodegeneration will be required. This calculation is performed using an online calculator (http://statulator.com/SampleSize/ss1P.html), where an expected proportion (sensitivity and specificity) of 50% was used as this yields the largest sample size. The diagnostic accuracy will be based on baseline parameters. To correct for potential dropout during follow-up, we increased the total number of participants to 200. Within these groups, participants will be divided into subgroups based on the clinical diagnosis. We aim to include a 50 participants per clinical diagnosis in order to obtain sufficient data to study secondary outcomes.

### Statistical analysis

All data will be stored in the online database Castor EDC with restricted access [30]. The final data will be exported to IBM SPSS Statistics (IBM, Armonk, NY, USA) for statistical analysis. Data from participants with missing data or data from withdrawn participants will be included in the statistical analysis. Threshold of statistical significance will be assumed equal to p < 0.05.

Receiver operating characteristic (ROC) analyses will be performed to estimate the capability of tear biomarkers to differentiate between individuals with neurodegeneration and those without neurodegeneration as determined by hippocampal atrophy. The area under the curve and diagnostic accuracy measures such as sensitivity, specificity, predictive values and likelihood rations (with the corresponding 95% confidence intervals) will be calculated to assess the discriminative capability of a biomarker. To study the correlation of biomarker levels in tears, blood, CSF and PET will be performed using Pearson or Spearman correlation. Each biomarker will be separately compared across three body fluids, and the Aβ region of interest SUVr from PET. Based on our pilot results, we expect negative correlations between tears and CSF (similar to blood and CSF) [17]. Diagnostic accuracy will also be determined for the combination of tear and retinal biomarkers. Linear scores will be derived from the total area or partial area under the ROC curve. Lastly, we will study the mean change of tear biomarkers over time and how this correlates with disease progression (based on neuropsychological evaluation, imaging and fluid biomarkers) with the use of (generalized) linear mixed models or repeated measures ANOVA. Post hoc analysis will reveal which groups and time points differ significantly from each other.

### Data management and monitoring

The personal data from participants will be handled confidentially, according to Good Clinical Practice guidelines (GCP). Medical history and associated data will be extracted from the electronic patient record. Data will be pseudonymized and stored in a certified online case report form tool (Castor EDC). Personal data that connects the patient to the participant number will be stored by the principal investigator for 15 year according to national guidelines. The study is monitored by the Clinical Trial Center Maastricht (CTCM) in order to assure patient rights and the accuracy of the reported data.

## Discussion

As the number of people affected by Alzheimer’s disease will grow in the upcoming years due to increased life expectancy, there is a need for an accurate, low-invasive and low-cost diagnostic test that could enable early diagnosis. Future therapies combined with earlier diagnosis can increase the chance for preserving cognitive functioning. When people with cognitive complaints are referred to a memory clinic, a biomarker test (PET scan or CSF puncture) is not always chosen, often due to the fact that they are expensive, invasive, or not available. Diagnosis with tear fluid could lower the bar to apply biomarker tests, since it is low-cost and less invasive. Additionally, tear fluid can be collected at the general practitioner where the patient presents first, enabling timely detection. Tear fluid collection also has the potential to be used for screening purposes, due to its minimally invasive nature.

One of the main strengths of our study is the wide range of information that will be available from the participants, ranging from neuropsychological to ophthalmic tests, which will thereby generate a deeply phenotyped cohort. Furthermore, this study combines measurements of tear and retinal biomarkers, which are both low-invasive methods that potentially complement each other. Potential limitations of our study are that participants may be lost for follow-up visits, due to declining to participate, emigration or study drop-out. Particularly patients with fast cognitive decline may be lost somewhere along the two year follow-up. Another limitation is that not all measurements and data will be available for all participants due to different standard procedures and available equipment per study location.

The TearAD study will be the first longitudinal study analyzing tear AD biomarkers in a large cohort. The first results of the TearAD study, expected by early 2025, will inform whether tear AD biomarkers have added value in the screening and diagnosis of AD.

### Study status

Recruitment of participants is ongoing. This started on the 24th of March 2021 and is expected to be completed by December 2023, after which participants will return for follow-up visits after 12 and 24 months. The latest version of the study protocol is version 2.0 (date: 16-05-2022).

## Data Availability

Not applicable.

## Abbreviations

AD: Alzheimer’s disease
ATN: Amyloid/Tau/Neurodegeneration
Amsterdam UMC: Amsterdam University Medical Center
Aβ: Amyloid-beta
CSF: Cerebral spinal fluid
CT: Computed tomography
ETDRS: Early treatment diabetic retinopathy study
GCP: Good clinical practice
NFT: Neurofibrillary tangles
NPA: Neuropsychological assessment
Maastricht UMC+: Maastricht University Medical Center
MCI: Mild cognitive impairment
MPOD: Macular pigment optical density
MRI: Magnetic resonance imaging
MTA: Medial temporal lobe atrophy
NIA-AA: National Institute on Ageing-Alzheimer’s Association
OCT: Optical coherence tomography
PET: Positron emission tomography
p-tau: Phosphorylated-tau
RNFL: Retinal nerve fiber layer
ROC: Receiver operating characteristic
SCD: Subjective cognitive decline
t-tau: Total-tau
